# The Association between Anomalous Self-experiences, Self-esteem and Depressive Symptoms in First Episode Schizophrenia

**DOI:** 10.3389/fnhum.2016.00557

**Published:** 2016-11-07

**Authors:** Elisabeth Haug, Merete G. Øie, Ole A. Andreassen, Unni Bratlien, Kristin L. Romm, Paul Møller, Ingrid Melle

**Affiliations:** ^1^Division of Mental Health, Innlandet Hospital TrustBrumunddal, Norway; ^2^Department of Psychology, University of OsloOslo, Norway; ^3^Research Department, Innlandet Hospital TrustBrumunddal, Norway; ^4^NORMENT K.G. Jebsen Centre for Psychosis Research, Oslo University Hospital and Institute of Clinical Medicine, University of OsloOslo, Norway; ^5^Division of Mental Health and Addiction, Oslo University HospitalOslo, Norway; ^6^Division of Mental Health and Addiction, Department of Mental Health Research and Development, Vestre Viken Hospital TrustLier, Norway

**Keywords:** schizophrenia, anomalous self-experiences, depression, self-esteem, first episode psychosis, childhood trauma, gender differences

## Abstract

**Background:** Anomalous self-experiences (ASEs) aggregate in schizophrenia spectrum disorders, but the relationship between ASEs, and depression has been studied to a limited extent. Lower self-esteem has been shown to be associated with depression in early psychosis. Our hypothesis is that ASEs in early phases of schizophrenia are linked to lower levels of self-esteem, which in turn is associated with depression.

**Aim:** The aim is to examine the relationship between ASEs, self-esteem and depression in first-episode schizophrenia spectrum disorders.

**Method:** ASEs were assessed in 55 patients with first-episode schizophrenia by means of the Examination of anomalous Self-Experience (EASE) instrument. Assessment of depression was based on the Calgary Depression Scale for Schizophrenia (CDSS). Self-esteem was measured using the Rosenberg Self-Esteem Scale (RSES). Symptom severity was assessed using the Structured Clinical Interview for the Positive and Negative Syndrome Scale (SCI-PANSS). Substance misuse was measured with the Drug Use Disorder Identification Test (DUDIT), and alcohol use was measured with the Alcohol Use Disorder Identification Test (AUDIT). Data on childhood adjustment were collected using the Premorbid Adjustment Scale (PAS). Data on childhood trauma were collected using the Norwegian version of the Childhood Trauma Questionnaire, short form (CTQ-SF).

**Results:** Analyses detected a significant association between current depression and ASEs as measured by the EASE in women, but not in men. The effect of ASEs on depression appeared to be mediated by self-esteem. No other characteristics associated with depression influenced the relationship between depression, self-esteem and ASEs.

**Conclusion:** Evaluating ASEs can assist clinicians in understanding patients' experience of self-esteem and depressive symptoms. The complex interaction between ASEs, self-esteem, depression and suicidality could be a clinical target for the prevention of suicidality in this patient group.

## Introduction

### Schizophrenia and anomalous self-experience

Studies show that anomalous self-experiences (ASEs) aggregate in schizophrenia spectrum disorders (Haug et al., 2012a; Nelson et al., 2013; Nordgaard and Parnas, 2014), and precede their onset (Parnas et al., 1998; Møller and Husby, 2000; Nelson et al., 2012). The sense of self (identity feeling) can be described on three hierarchical but interconnected levels: the narrative, the reflective, and the prereflective identity level (Sass and Parnas, 2003). The narrative- or social self refers to certain explicit characteristics, like personality traits and the overt narratives of the person; whereas the reflective self is the awareness of a stable “I” over time and situations. The prereflective self is the most basic level of self-awareness, implicit, preverbal, and inseparable from subjective experience *per se*. This prereflective self-awareness, also described as the basic self, is a necessary basis for the other two levels.

ASEs are subtle disturbances of the prereflective self, affecting the person's deepest sense of being, the experience of him- or herself as a vital subject, naturally immersed in the world, and the sense of continuity and coherence in self-experience (Sass and Parnas, 2003). ASEs include certain and subtle forms of depersonalization, anomalous experiences of cognition and stream of consciousness, self-alienation, pervasive difficulties in grasping familiar and taken-for-granted meanings, unusual bodily feelings and existential reorientation (Parnas et al., 2005). In schizophrenia, ASEs are believed to underpin and generate several symptom dimensions such as positive, negative, and disorganized psychotic symptoms (Sass and Parnas, 2003). An earlier study also found a link between ASEs and suicidality among patients with schizophrenia (Skodlar and Parnas, 2010), and we have in previous reports from the current study also shown that ASEs are linked to suicidality (Haug et al., 2012b) in addition to a longer duration of untreated psychosis (DUP) (Haug et al., 2015b), social dysfunction (Haug et al., 2014) and childhood trauma, the latter however only in women (Haug et al., 2015a). We have here observed an association between ASEs and depressive symptoms (Haug et al., 2012b, 2015a).

### Schizophrenia and depressive symptoms

Depressive symptoms are common in patients with schizophrenia spectrum disorders (Birchwood et al., 2000) and is particularly prevalent in first episode psychosis (Romm et al., 2010). There are several possible pathways to depressive symptoms in schizophrenia (Birchwood, 2003; Skodlar, 2009). The first possibility is that depressive and psychotic symptoms are parts of two different disorders that co-occur due to overlapping risk factors (such as social difficulties and childhood maltreatment). Another possibility is that it is a psychological reaction to the psychosis, either through its implications for social status (Birchwood, 2003) or as a reaction to the experience of psychological deficits (Liddle et al., 1993). Difficult childhood experiences, such as childhood loss and social marginalization could also contribute to a cognitive vulnerability that is accompanied by a negative view of the person him/herself and toward others (Greenberg et al., 1992; Garety et al., 2001; Birchwood, 2003). Finally, it could be a core dimension of the psychosis in line with negative symptoms (Upthegrove et al., 2016)

### Anomalous self-experience and depressive symptoms

The relationship between disturbances in basic self-awareness, i.e., ASEs, and depressive symptoms has been studied to a limited extent. Yon and colleagues found that subjective experience, measured by the Frankfurt Complaint Questionnaire, was separate and distinct from the objective symptomatology in schizophrenia (Yon et al., 2005). Another study found that depressed patients with schizophrenia showed significantly higher levels of basic symptoms as measured by Bonn Scale for the Assessment of Basic Symptoms, a concept related to ASEs (Maggini and Raballo, 2006). The aim of the current study is to explore this association in more detail. Skodlar suggested that feelings of inferiority could serve as link between ASEs and suicidality; i.e., a version of the reaction hypothesis. In line with this, we chose to examine if self-esteem mediated the association between ASEs and depressive symptoms.

### Broadening the scope with self-esteem

Self-esteem was introduced in this study to shed a broader light on this symptom complex. Self-esteem reflects a person's overall subjective emotional evaluation of his or her own worth and is the positive or negative evaluations of the self, while the self-concept is what we think about the self (Smith and Mackie, 2007). Maslow included positive self-esteem in his hierarchy of human needs. He described two variants of “esteem”: the need for respect from others, and the need for self-respect (Maslow, 1987). People need both these forms of “esteem” to grow as a person and achieve self-actualization (Maslow, 1987). There are different factors that can influence self-esteem. Self-esteem is usually regarded as an enduring personality characteristic. Genetic factors that help shape overall personality can play a role, but it is often our life experiences that form the basis of overall self-esteem. Models of global self-esteem suggest that it is both a trait and a state measure (Crocker and Wolfe, 2001). Rosenberg made distinctions between *baseline* instability, i.e., long- term fluctuations in self-esteem that gradually changes over a longer period of time, and *barometric* instability, which reflects the short term fluctuations in ones contextually based global self-esteem (Rosenberg, 1986). It is the person's interpretation of the event or circumstance, and its relevance to his or her contingencies of self-worth, that determines both if and how strongly it will affect state self-esteem (McFarl and Ross, 1982; Crocker and Wolfe, 2001).

Lower self-esteem has repeatedly been shown to be associated with depressive symptoms, also in patients with early psychosis (Karatzias et al., 2007) where it is viewed as both a possible cause—and a possible consequence of psychotic symptoms (Karatzias et al., 2007; Romm et al., 2011). The two concepts of self-esteem and self-disturbances are based in very different theoretical frameworks. Self-esteem can however be seen as related to the narrative- or social level of selfhood.

Our hypothesis for the current study is thus that ASEs in early phases of schizophrenia are linked to poor self-esteem, which in turn is associated with depressive symptoms. The inclusion of self-esteem may contribute to more knowledge about the phenomenology of depressive symptoms in schizophrenia, and may thus have implications for therapeutic approaches to a condition that is accompanied with considerable suffering and risk of suicide.

In previous studies men have been showing lower levels of depression and higher levels of self-esteem than women (Thorup et al., 2007; Romm et al., 2011), and in previous reports from the current study we have shown that ASEs are linked childhood trauma only in women (Haug et al., 2015a). Thus, there is a possibility of real gender difference in this area.

## Materials and methods

### Design and sample

The current study is part of the Norwegian Thematically Organized Psychosis (TOP) study (Romm et al., 2010). Inclusion criteria were age 18–65 years, and being consecutive in- or outpatient referred to first adequate treatment for a psychotic disorder that is a DSM-IV diagnosis of schizophrenia (schizophrenia, schizophreniform disorder, and schizoaffective disorder). Exclusion criteria were the presence of brain injury, neurodegenerative disorder, or mental retardation. The patients were required not be so overtly psychotic that they had problems participating in a lengthy interview. Patients with concurrent substance use disorders were not excluded, but had to demonstrate at least 1 month without substance use, or clear signs that the psychotic disorder had started before the onset of significant substance use (i.e., did not meet the criteria for substance induced psychotic disorder). The sample includes all consecutively identified first episode patients from all treatment facilities in two Norwegian counties (Hedmark and Oppland) with a county-wide population of 375,000 people. During 2008 and 2009 a total of 44 patients with schizophrenia spectrum disorders were coming to their first adequate treatment (i.e., not having previously received adequate antipsychotic medication in adequate doses for 12 weeks, or until remission); some had not initiated treatment at first evaluation. To enhance statistical power we additionally included 11 patients enrolled in a related study of young patients with psychosis born in 1985/86 (Bratlien et al., 2014, 2015). They were in the early phases of treatment, with an even shorter DUP than the strict first treatment patients, and met the same inclusion and exclusion criteria, except for the strict definition of first treatment.

All participants gave written, informed consent to participate in accordance with the Declaration of Helsinki. The study was approved by the Regional Committee for Medical Research Ethics and the Norwegian Data Inspectorate.

### Clinical assessments

Diagnoses were ascertained by two researchers who were also experienced psychiatrists (EH and UB) using the Structural Clinical Interview for the Diagnostic and Statistical Manual of Mental Disorders, fourth edition (SCID-IV) (Amercan Psychiatric Association, 1994).

ASEs were assessed using the Examination of Anomalous Self-Experience (EASE) manual (Parnas et al., 2005), a 30–90 min interview comprising five domains: (1) Cognition and stream of consciousness. (2) Self-awareness and presence. (3) Bodily experiences. (4) Demarcation/transitivism. (5) Existential reorientation. This represents a wide variety of ASEs condensed into 57 main items and scored on a 5-point Likert scale (0–4), in which 0 = absent; 1 = questionably present; 2 = definitely present, mild; 3 = definitely present, moderate; 4 = definitely present, severe. For the purpose of the analyses, the resulting scores were dichotomized into 0 (absent or questionably present) and 1 (definitely present, all severity levels). ASEs are not considered to be discrete symptoms but rather interconnected aspects of a full gestalt. There are thus considerable overlap between single items and domains, and both items and domains are statistically highly inter-correlated. We have thus used the total EASE score in the analyses. The questions about ASEs in the EASE are not focused on a specific time period but capture life-time experiences of ASEs. EH was trained by one of the main authors of the EASE (PM) and conducted all the interviews. The inter-rater reliability (IRR) of the EASE, including in the current study, has been found to be very good (Møller et al., 2011; Nelson et al., 2012; Raballo and Parnas, 2012).

Assessment of depressive symptoms was based on the Calgary Depression Scale for Schizophrenia (CDSS) (Addington et al., 1990). Self-esteem was measured using the Rosenberg Self-Esteem Scale (RSES) (Rosenberg, 1965). This is a 10 item self-administered questionnaire with a 4-point Likert-type response set, ranging from strongly disagree to strongly agree on statements about their self-esteem and self-deprecation. RSES is validated and used in several studies with psychotic patients (Torrey et al., 2000). Symptom severity was assessed using the Structured Clinical Interview for the Positive and Negative Syndrome Scale (SCI-PANSS) (Kay et al., 1987). We have in our analyses used the Wallwork/Fortgang five-factor model for PANSS (Wallwork et al., 2012), which is recommended for describing symptoms in patients with first episode psychosis. Insight was assessed by PANSS item G 12 (insight). G12 is a global measure of insight used in many studies of psychotic patients. Substance misuse was measured with the Drug Use Disorder Identification Test (DUDIT) (Berman et al., 2007), and alcohol use was measured with the Alcohol Use Disorder Identification Test (AUDIT) (Saunders et al., 1993). Data on childhood adjustment were collected using the Premorbid Adjustment Scale (PAS) (Cannon-Spoor et al., 1982). Data on childhood trauma were collected using the Norwegian version of the Childhood Trauma Questionnaire, short form (CTQ-SF) (Bernstein et al., 2003). This is a 28-item self-report inventory, developed and validated based on the original 70-item version (Bernstein et al., 1997), that provides a relatively short screening of maltreatment experiences before the age of 18. It comprises 28 items, yielding scores on five subscales of trauma: physical abuse, sexual abuse, emotional abuse, emotional neglect, and physical neglect.

Both researchers involved in the clinical assessments (EH and UB) completed the TOP study group's training and reliability program with SCID training based on- and supervised by the UCLA training program (Ventura et al., 1998). For DSM-IV diagnostics, mean overall kappa for the standard diagnosis of training videos for the study as a whole was 0.77, and mean overall kappa for a randomly drawn subset of study patients was also 0.77 (95% CI 0.60–0.94). Intra Class Coefficients (ICC 1.1) for the other scales were: PANSS positive subscale 0.82 (95% CI 0.66–0.94), PANSS negative subscale 0.76 (95% CI 0.58–0.93), and PANSS general subscale 0.73 (95% CI 0.54–0.90).

### Statistical analysis

All analyses were performed with the statistical package SPSS, version 21.0 (SPSS, Chicago, IL). Mean and standard deviations are reported for continuous variables and percentages for categorical variables. Since DUP had a markedly skewed distribution, median, and range values are reported and a transformation into its natural logarithm was used in parametric analyses. We first examined bivariate associations between depressive symptoms, ASEs and self-esteem, respectively using Pearson correlations, and then the association between depressive symptoms and ASEs after controlling for levels of self-esteem, using multiple linear regression analysis. We used the Sobel test to evaluate mediation. We then went on to examine possible confounders of these associations using a series of multiple linear regression analyses, correcting for patient characteristics associated with depressive symptoms one at a time (since the sample size did not allow for more than four to five variables in the equation). There were no interaction effects.

## Results

Table 1 presents the sociodemografic and clinical features of the sample. The mean EASE total score was 25.5, which is at the same level as other studies of ASEs in schizophrenia. The mean CDSS score was 9.1, indicating high levels of depressive symptoms. We found a statistically significant positive association between ASEs (EASE total score) and depressive symptoms (*r* = 0.356 *p* = 0.008) and a negative association with self-esteem (*r* = −0.361 *p* = 0.007), indicating that high levels of ASEs were associated with high levels of depressive symptoms and low self-esteem. EASE domain 1, 3, and 4 were significantly correlated with depressive symptoms, while EASE domain 1, 2, and 3, were significantly correlated with self-esteem (data not shown). The main analyses of the current paper focus on the EASE total score. As expected, we also found a strong and significant negative association between depressive symptoms and self-esteem (*r* = −0.761, *p* < 0.0001). In a multiple linear regression analysis the effect of ASEs on depressive symptoms was no longer significant after correcting for levels of self-esteem, indicative of a mediation effect (Table 2). This was supported by a significant positive Sobel test (*p* = 0.01).

**Table 1:** **Demographic and clinical characteristics**.

**Number of patients**	**55**
**DEMOGRAPHICS**	
Male gender, n (%)	28 (51)
Age years, mean (SD)	25.2 (7.3)
DUP^a^ weeks, median (range)	122 (2–1560)
**PREMOBID ADJUSTMENT^b^**	
Childhood, mean (SD)	0.3 (0.2)
Early adulthood, mean (SD)	0.4 (0.2)
**SUBSTANCE USE**	
Alchohol^c^, mean (SD)	9.1 (8.8)
Drugs^d^, mean (SD)	2.9 (7.8)
**SYMPTOMS**	
Depressive symptoms^e^, mean (SD)	9.1 (6.0)
ASEs^f^, mean (SD)	25.5 (9.7)
Self-esteem^g^, mean (SD)	21.4 (6.2)
**PANSS^h^**	
Positive symptoms, mean (SD)	13.9 (5.6)
Negative symptoms, mean (SD)	14.1 (6.7)
Disorganization symptoms, mean (SD)	6.6 (3.2)
Depressive symptoms, mean (SD)	9.7 (3.3)
Excitement symptoms, mean (SD)	6.4 (2.1)
Childhood trauma^i^, mean (SD)	47.2 (18.8)

a *Duration of Untreated Psychosis*.

b *PAS (Premorbid Adjustment Scale)*.

c *AUDIT (Alcohol Use Disorder Identification Test) total score*.

d *DUDIT (Drug Use Disorder Identification Test) total score*.

e *CDSS (Calgary Depression Scale for Schizophrenia) total score*.

f *EASE (Examination of Anomalous Self-Experience) total score*.

g *RSES (Rosenberg Self-Esteem Scale) total score*.

h *Wallwork/Fortgang five-factor model for PANSS (Positive and Negative Syndrome Scale)*.

i *CTQ (Childhood Trauma Questionnaire) total score*.

**Table 2:** **Hierarchical regression analysis with depressive symptoms as the dependent variable and ASEs and self-esteem as independent variables, demonstrating mediating effects of self-esteem**.

**Dependent variable: Depressive symptoms^a^**	**B**	***p***	**95% CI**
ASEs^b^	0.356^*^	0.008	0.060 to 0.377
**Dependent variable: Depressive symptoms^a^**	**B**	***p***	**95% CI**
Self-esteem^c^	−0.761^**^	< 0.001	−0.896 to −0.555
**Dependent variable: Depressive symptoms^a^**	**B**	***p***	**95% CI**
Self-esteem^c^	−0.727^**^	< 0.001	−0.876 to −0.510
ASEs^b^	0.093	0.337	−0.061 to 0.175

a *CDSS (Calgary Depression Scale for Schizophrenia) total score*.

b *EASE (Examination of Anomalous Self-Experiences) total score*.

c *RSES (Rosenberg Self-Esteem Scale) total score*.

* *Correlation is significant at the 0.05 level (2-tailed)*.

** *Correlation is significant at the 0.001 level (2-tailed)*.

We then examined the association to other variables with a putative effect on depressive symptoms. We found a statistically significant association between depressive symptoms and childhood trauma, the PANSS negative- and disorganized sub scales, drug use and female gender; but not with other PANSS sub scales, insight, DUP, premorbid adjustment or alcohol use (Table 3). In the ensuing multiple linear regression analysis, we explored if the characteristics associated with depressive symptoms influenced the relationship between depressive symptoms, self-esteem and ASEs. Of these variables the scores on the PANSS negative- and disorganized sub scales and the childhood trauma had an independent effect on depressive symptoms when entered in the multiple linear regression analyses; but without influencing the relationship between depressive symptoms, self-esteem and ASEs.

**Table 3:** **Correlations**.

	**Depressive symptoms^a^**
**Gender**	
Pearson correlation	0.333^*^
Sig. (2-tailed)	0.013
**Duration of untreated psychosis**^b^	
Pearson correlation	0.102
Sig. (2-tailed)	0.460
**Premorbid adjustment**^c^	
**Childhood**
Pearson correlation	0.128
Sig. (2-tailed)	0.350
**Early adulthood**
Pearson correlation	−0.054
Sig. (2-tailed)	0.69
**Alcohol use**^d^	
Pearson correlation	0.105
Sig. (2-tailed)	0.447
**Drug use**^e^	
Pearson correlation	0.286^*^
Sig. (2-tailed)	0.034
**PANSS**^f^	
**Positive symptoms**	
Pearson correlation	0.168
Sig. (2-tailed)	0.219
**Negative symptoms**	
Pearson correlation	−0.289^*^
Sig. (2-tailed)	0.032
**Disorganization symptoms**	
Pearson correlation	−0.343^*^
Sig. (2-tailed)	0.010
**Depressive symptoms**
Pearson correlation	0.634^**^
Sig. (2-tailed)	0.000
**Excitement symptoms**
Pearson correlation	0.102
Sig. (2-tailed)	0.459
**Insight**^g^	
Pearson correlation	−0.154
Sig. (2-tailed)	0.262
**Childhood trauma**^h^	
Pearson correlation	0.568^**^
Sig. (2-tailed)	< 0.001

a *CDSS (Calgary Depression Scale for Schizophrenia) total score*.

b *Ln DUP*.

c *PAS (Premorbid Adjustment Scale)*.

d *AUDIT (Alcohol Use Disorder Identification Test) total score*.

e *DUDIT (Drug Use Disorder Identification Test) total score^f^ Wallwork/Fortgang five-factor model for PANSS (Positive and Negative Symptom Scale)*.

f *Wallwork/Fortgang five-factor model for PANSS (Positive and Negative Syndrome Scale)*.

g *PANSS item g12*.

h *CTQ (Childhood Trauma Questionnaire) total score*.

* *Correlation is significant at the 0.05 level (2-tailed)*.

** *Correlation is significant at the 0.001 level (2-tailed)*.

Investigating males and females separately we found a strong and significant negative association between depressive symptoms and self-esteem in both men and women (data not shown). Further we found that women had more depressive symptoms and lower levels of self-esteem than men, with a highly statistically significant association between ASEs, self-esteem (*r* = −0.481, *p* = 0.011) and depressive symptoms (*r* = 0.488, *p* = 0.010) in women, but not in men. In a multiple linear regression analysis the effect of ASEs on depressive symptoms in women was no longer significant after correcting for levels of self-esteem, indicative of a mediation effect (Table 4). This was supported by a significant positive Sobel test (*p* = 0.01). We then examined the association to other variables with a putative effect on depressive symptoms in women. We found a statistically significant association between depressive symptoms and childhood trauma, the PANSS negative- and disorganized sub scales, DUP, and premorbid childhood adjustment (data not shown). Of these variables only the scores on the childhood trauma score had an independent effect on depressive symptoms when entered in the multiple linear regression analyses; but without influencing the relationship between depressive symptoms, self-esteem and ASEs.

**Table 4:** **Women: Hierarchical regression analysis with depressive symptoms as the dependent variable and ASEs and self-esteem as independent variables, demonstrating mediating effects of self-esteem in women**.

**Dependent variable: Depressive symptoms^a^**	***B***	***p***	**95% CI**
ASEs^b^	0.327^*^	0.010	0.086 to 0.569
**Dependent variable: Depressive symptoms**^a^	***B***	***p***	**95% CI**
Self-esteem^c^	−0.790^**^	< 0.001	−1.035 to −0.544
**Dependent variable: Depressive symptoms**^a^	***B***	***p***	**95% CI**
Self-esteem^c^	−0.725^**^	< 0.001	−1.006 to −0.444
ASEs^b^	0.091	0.334	−0.099 to 0.281

a *CDSS (Calgary Depression Scale for Schizophrenia) total score*.

b *EASE (Examination of Anomalous Self-Experiences) total score*.

c *RSES (Rosenberg Self-Esteem Scale) total score*.

* *Correlation is significant at the 0.05 level (2-tailed)*.

** *Correlation is significant at the 0.001 level (2-tailed)*.

## Discussion

Our main finding in the present study is that the association between ASEs and depressive symptoms in women appears to be mediated by self-esteem. We have previously reported an association between ASEs and depressive symptoms (Haug et al., 2012b). This is in line with an earlier study that found that depressed patients showed significantly higher levels of basic symptoms measured by BSABS (Gross et al., 1987) a construct that is related to ASEs (Maggini and Raballo, 2006). As far as we know, our current study is the first study on the association between ASEs and self-esteem. These results are however in line with the hypothesis presented by Skodlar and associates (Skodlar et al., 2008; Skodlar and Parnas, 2010), where the authors suggested that the effect of ASEs on depressive symptoms and suicidality was mediated by specific feelings of inferiority that could be an aspect of low self-esteem. ASEs also include disturbances of the basic self, to the extreme extent that the person feels as if not existing or not being human. These are obviously experiences that accelerate the feeling of worthlessness. It is also in line with observations from Liddle and colleagues, that experience of psychological deficits in schizophrenia was associated with depression (Liddle et al., 1993), where ASEs in this context could be experienced as- or resemble psychological deficits. The results thus primarily support the hypothesis that depression is a reaction to the psychotic illness. The independent effect of childhood trauma also supports the notion of common predictors to depression and to psychosis.

The current study is cross-sectional and can thus not say anything directly about the direction of association. However, the ASEs are subtle and relatively stable disturbances of the most basic level of self-awareness, depressive symptoms are more fluid and state-like phenomena while self-esteem can be viewed as both a trait and a state phenomenon (Crocker and Wolfe, 2001). Our interpretation of the findings is thus that ASEs contribute to lower self-esteem, which in turn increases the risk of depressive symptoms.

The association between ASEs, self-esteem and depression appeared to be carried by the female part of the sample. Men showed lower levels of depression and higher levels of self-esteem than women in line with previous studies (Thorup et al., 2007; Romm et al., 2011), while the direction of associations were the same for both genders with no interaction effects. We thus interpret the differences in statistically significant associations as mainly based in lower statistical power in the male part of the sample. The possibility of real gender differences in this area should however be kept in mind and included in further investigations.

In addition to high levels of depressive symptoms, the current sample was also characterized by a long median DUP. Long DUP has previously been shown to be associated with depressive symptoms (Marshall et al., 2005). However, in the current study we did find significant correlations between DUP and depressive symptoms only in women, but it does not appear as if the association between ASEs and depressive symptoms was mediated by DUP. The presence of childhood trauma was associated with both ASEs and depressive symptoms, but did not appear to mediate the relationship.

The current findings suggest that evaluating ASEs can assist clinicians in understanding patients' experience of self-esteem and depressive symptoms. The ASE perspective has turned out to be fruitful in clinical settings, and for therapeutic interventions; as it gives an experience of comprehension and meaning back to the patients by making bizarre experiences subjectively understandable and thus possible to communicate to others. Suicidality is a major complication in the early phases of schizophrenia and is associated with both ASEs and depressive symptoms (Haug et al., 2012b). Thus, the complex interaction between ASEs, self-esteem, depressive symptoms, and suicidality could be a clinical target for the prevention of suicidality in this patient group.

More knowledge about this may also have implications for other treatment approaches, including CBT schema therapy targeting depressive symptoms. Birchwood postulated that childhood trauma and psychosis-like experiences in early adolescence, ASEs associated phenomena, may contribute to cognitive schemas characterized by negative self-evaluation that predispose to depressive symptoms as a reaction to psychosis (Birchwood, 2003). This is in line with our previously reported association between childhood trauma and ASEs in women (Haug et al., 2015a). The current findings however indicate that childhood trauma also influence depressive symptoms through other pathways than ASEs and self-esteem.

### Strengths

We included patients in the early phase of the treated course of the disorder, thereby minimizing potential confounding effects such as selection of non-responders and chronicity that might impact on the assessment of ASEs, self-esteem, and depressive symptoms. The Norwegian mental health care offers public mental health care to all individuals with mental illness within a given catchment area. Because of the next-to absence of private mental health care in Norway, the sample is not biased for socioeconomic class. The study included all consecutive in- or outpatients referred to treatment for a psychotic disorder in two neighboring Norwegian counties in a defined time period, and the participants are thus highly representative of the patient group.

### Limitations

The correlational nature of this study gives neither firm conclusions about the direction of associations, nor about causality. High levels of ASEs and low levels of self-esteem could also be influenced by recall bias among patients with high levels of depressive symptoms. Previous studies of the temporal relationship between these factors however indicate that the subjective experience of psychological deficits in schizophrenia patients with depressive episodes is high, even when they are not depressed (Liddle et al., 1993). The size of the study sample imposes limits on statistical approaches to study complex interactions. To combine data intended to tap different levels of the self/ (self-esteem and self-disturbance) raises conceptual dilemmas. Self-esteem is mainly conceived as a fully conscious social/narrative level, whereas self-disturbance in the present context refers to a pre-reflective, pre-conscious level. While the scale used to measure self-esteem is validated in groups with psychotic disorders, it is not fully clear if the instrument take sufficiently into account the structural distinctiveness of psychotic consciousness in persons with severe self-disorders. Results should thus be interpreted with caution.

## Author contributions

EH, IM, MØ, and PM planned the current study, and OA and KR contributed to the study design. EH and UB contributed to data collection. EH conducted the statistical analyses and also wrote the first draft of the manuscript, while IM contributed to the analyses. All the authors contributed to the interpretation of the data and participated in critical revision of manuscript drafts, approved the final version and agreed to be accountable for all aspects of the work in ensuring that questions related to the accuracy or integrity of any part of the work are appropriately investigated and resolved.

## Funding

Funding for this study was provided by Innlandet Hospital Trust (grant number 150229) and the Regional Health Authority of South-Eastern Norway (grant numbers, 2006258, 2008058, and 2011085). The funding sources had no further role in study design; in the collection, analysis and interpretation of data; in the writing of the report; and in the decision to submit the paper for publication.

### Conflict of interest statement

The authors declare that the research was conducted in the absence of any commercial or financial relationships that could be construed as a potential conflict of interest.
